# Supplementary material for: Network properties determine neural network performance
Source: Nat Commun. 2024 Jul 8;15:5718. doi: 10.1038/s41467-024-48069-8 (PMC11231255; doi:10.1038/s41467-024-48069-8)
Supplement: Supplementary file 1 — Supplementary Information [file 41467_2024_48069_MOESM1_ESM.pdf]

Supplemental Information

Network Properties Determine Neural Network Performance

Chunheng Jiang,[1,2,*] Zhenhan Huang,[1,2,*] Tejaswini Pedapati,[3] Pin-Yu Chen,[3] Yizhou Sun,[4] and Jianxi Gao[1,2,†]

[1]Network Science and Technology Center, Rensselaer Polytechnic Institute, NY USA

[2]Department of Computer Science, Rensselaer Polytechnic Institute, NY USA

[3]IBM Thomas J. Watson Research Center, Yorktown Heights, NY USA

[4]Department of Computer Science, University of California, Los Angeles, CA USA

---

*Chunheng Jiang and Zhenhan Huang contribute equally to this work.

†Correponding author: gaoj8@rpi.edu

Contents

Supplementary Note 1. Error Gradients 3

Supplementary Note 2. Weighted Degrees of G_B 4

Supplementary Note 3. Derivation of Adjacency Matrix P of G_B 7

Supplementary Note 4. Proof of Theorem 1 8

Supplementary Note 5. Running Time Comparison 8

Supplementary Note 6. Mean-Field Approach 9

Supplementary Note 7. Computational cost of the proposed framwork 10

Supplementary Note 8. Comparison using standard nas benchmarks 10

A. LC based predictors as baselines 10

B. Training-Free NAS metrics as baselines 11

SUPPLEMENTARY REFERENCES 11

Supplementary Note 1. ERROR GRADIENTS

Let G_A be an MLP. To understand the learning mechanism, we take a sample $\boldsymbol{x}$ with label $\boldsymbol{y}$, and go through the entire training procedure, including a forward pass FP and a backward pass BP. To be convenient, we rewrite $\boldsymbol{z}^{(0)} = \boldsymbol{x}$ as the inputs, let $\boldsymbol{z}^{(1)}$ and $\boldsymbol{z}^{(L-1)}$ be the activations of the first and the last hidden layers, respectively, and let $\boldsymbol{z}^{(L)}$ be the outputs. The MLP is a parameterized model $\hat{\boldsymbol{y}} = \boldsymbol{z}^{(L)} = F_{\boldsymbol{w}}(\boldsymbol{x})$ with $\boldsymbol{w} = (W^{(1)}, W^{(2)}, \ldots, W^{(L)})$, where $W^{(\ell)}$ is the weight matrix of synaptic connections from layer $\ell - 1$ to layer ℓ, and $1 \leq \ell \leq L$. Suppose there are n_ℓ neurons on layer ℓ, $W^{(\ell)}$ has the size $n_\ell \times n_{\ell-1}$. The inputs $\boldsymbol{x}$ are fed into MLP, after a forward pass from layer 1 down to layer $L - 1$ and layer L. Each neuron receives a cumulative input signal from the previous layer, and sends an activated signal to a downstream layer. Let σ_ℓ be the activation function of layer ℓ, $a_i^{(\ell)} = \boldsymbol{w}_i^{(\ell)T} \boldsymbol{z}^{(\ell-1)}$ be the pre-activation value of neuron i on layer ℓ, we have $z_i^{(\ell)} = \sigma_\ell(a_i^{(\ell)})$ with $w_i^{(\ell)}$ being the ith row of $W^{(\ell)}$, $1 \leq i \leq n_\ell$. The output activation function σ_L is generally a softmax function, i.e. $z_i^{(L)} = \exp\{a_i^{(L)}\} / \sum_j \exp\{a_j^{(L)}\}$, and $\boldsymbol{z}^{(L)}$ is a probability distribution over n_L classes, i.e. $\mathbf{1}^T \boldsymbol{z}^{(L)} = 1$.

With the predictions $\boldsymbol{z}^{(L)}$ and the ground truth $\boldsymbol{y}$, we can calculate the prediction error $\mathcal{C}(\boldsymbol{z}^{(L)}, \boldsymbol{y})$, which is often a cross entropy loss, i.e. $\mathcal{C}(\boldsymbol{z}^{(L)}, \boldsymbol{y}) = -\sum_i y_i \log z_i^{(L)}$. To minimize $\mathcal{C}$, BP is applied, and the weights $\boldsymbol{w}$ are updated backward, from the output layer up to the first hidden layer.

Now we derive the gradients for a close examination. First, we get the derivatives of $\mathcal{C}$ w.r.t $\boldsymbol{z}^{(L)}$ and $W^{(L)}$. Because $z_i^{(L)} = \exp\{a_i^{(L)}\} / \sum_j \exp\{a_j^{(L)}\}$, we get the gradient of the output $z_k^{(L)}$ w.r.t $w_{ij}^{(L)}$:

$$\partial z_k^{(L)} / \partial w_{ij}^{(L)} = z_k^{(L)} (\delta_{ki} - z_i^{(L)}) z_j^{(L-1)}, \tag{S1}$$

where $\delta_{ki} = 1$ if $k = i$, otherwise $\delta_{ki} = 0$.

On layer L, we get the derivatives of $\mathcal{C}$ w.r.t $\boldsymbol{z}^{(L)}$ and $W^{(L)}$:

$$\begin{aligned} \frac{\partial \mathcal{C}}{\partial z_i^{(L)}} &= -\frac{y_i}{z_i^{(L)}}, \\ \frac{\partial \mathcal{C}}{\partial w_{ij}^{(L)}} &= \sum_k \frac{\partial \mathcal{C}}{\partial z_k^{(L)}} \frac{\partial z_k^{(L)}}{\partial w_{ij}^{(L)}} = (z_i^{(L)} - y_i) z_j^{(L-1)}. \end{aligned} \tag{S2}$$

Then, we examine layer $L - 1$. The activation function σ_{L-1} associates to a pair of neurons $u_i^{(L-1)}$ on layer $L - 1$ and $u_j^{(L-2)}$ on layer $L - 2$ with a unique connection weight $w_{ij}^{(L-1)}$. Since $z_i^{(L)} = \exp\{a_i^{(L)}\} / \sum_j \exp\{a_j^{(L)}\}$ and $z_i^{(L-1)} = \sigma_{L-1}(a_i^{(L-1)})$, we get $\partial z_k^{(L)} / \partial z_i^{(L-1)} = z_k^{(L)} (w_{ki}^{(L)} -$

$\sum_j z_j^{(L)} w_{ji}^{(L)}) = z_k^{(L)}(w_{ki}^{(L)} - w_{*i}^{(L)T}\boldsymbol{z}^{(L)})$, and $\partial z_i^{(L-1)}/\partial w_{ij}^{(L-1)} = z_j^{(L-2)}\sigma'_{L-1,i}$. The derivatives of $\mathcal{C}$ are

$$\begin{aligned}\frac{\partial \mathcal{C}}{\partial z_i^{(L-1)}} &= \sum_k \frac{\partial \mathcal{C}}{\partial z_k^{(L)}} \frac{\partial z_k^{(L)}}{\partial z_i^{(L-1)}} = \sum_k y_k(w_{*i}^{(L)T}\boldsymbol{z}^{(L)} - w_{ki}^{(L)}) = w_{*i}^{(L)T}(\boldsymbol{z}^{(L)} - \boldsymbol{y}),\\ \frac{\partial \mathcal{C}}{\partial w_{ij}^{(L-1)}} &= \frac{\partial \mathcal{C}}{\partial z_i^{(L-1)}} \frac{\partial z_i^{(L-1)}}{\partial w_{ij}^{(L-1)}} = w_{*i}^{(L)T}(\boldsymbol{z}^{(L)} - \boldsymbol{y}) z_j^{(L-2)}\sigma'_{L-1,i}.\end{aligned} \tag{S3}$$

On layer ℓ, where $1 \le \ell \le L-2$, we get $\partial z_k^{(\ell+1)}/\partial z_i^{(\ell)} = w_{ki}^{(\ell+1)}\sigma'_{\ell+1,k}$ and

$$\begin{aligned}\frac{\partial \mathcal{C}}{\partial z_i^{(\ell)}} &= \sum_k \frac{\partial \mathcal{C}}{\partial z_k^{(\ell+1)}} \frac{\partial z_k^{(\ell+1)}}{\partial z_i^{(\ell)}} = \sum_k \frac{\partial \mathcal{C}}{\partial z_k^{(\ell+1)}} w_{ki}^{(\ell+1)}\sigma'_{\ell+1,k},\\ \frac{\partial \mathcal{C}}{\partial w_{ij}^{(\ell)}} &= \frac{\partial \mathcal{C}}{\partial z_i^{(\ell)}} \frac{\partial z_i^{(\ell)}}{\partial w_{ij}^{(\ell)}} = \frac{\partial \mathcal{C}}{\partial z_i^{(\ell)}} z_j^{(\ell-1)}\sigma'_{\ell,i},\end{aligned} \tag{S4}$$

according to the relations $z_i^{(\ell+1)} = \sigma_{\ell+1}(a_i^{(\ell+1)})$ and $z_i^{(\ell)} = \sigma_\ell(a_i^{(\ell)})$.

Let $\boldsymbol{\delta}^{(\ell)} = [\partial\mathcal{C}/\partial z_1^{(\ell)}, \cdots, \partial\mathcal{C}/\partial z_{n_\ell}^{(\ell)}]^T \in \mathcal{R}^{n_\ell}$, $\boldsymbol{\sigma}'_\ell = [\sigma'_{\ell,1}, \cdots, \sigma'_{\ell,n_\ell}]^T \in \mathcal{R}^{n_\ell}$, $1 \le \ell \le L$. We can write the gradients in a dense form:

$$\begin{aligned}\boldsymbol{\nabla}_{W^{(L)}} &= (\boldsymbol{z}^{(L)} - \boldsymbol{y})\boldsymbol{z}^{(L-1)T},\\ \boldsymbol{\delta}^{(L-1)} &= W^{(L)T}(\boldsymbol{z}^{(L)} - \boldsymbol{y}),\\ \boldsymbol{\nabla}_{W^{(L-1)}} &= (\boldsymbol{\delta}^{(L-1)} \odot \boldsymbol{\sigma}'_{L-1})\boldsymbol{z}^{(L-2)T},\\ \boldsymbol{\delta}^{(\ell)} &= W^{(\ell+1)T}(\boldsymbol{\delta}^{(\ell+1)} \odot \boldsymbol{\sigma}'_{\ell+1}),\\ \boldsymbol{\nabla}_{W^{(\ell)}} &= (\boldsymbol{\delta}^{(\ell)} \odot \boldsymbol{\sigma}'_\ell)\boldsymbol{z}^{(\ell-1)T}.\end{aligned} \tag{S5}$$

Supplementary Note 2. WEIGHTED DEGREES OF G_B

We examine three hidden layers $\{\ell-1, \ell, \ell+1\}$ of G_A and three neurons on these layers $\{j, i, k\}$. Let $w_{ki}^{(\ell+1)}$ connects the neuron k on layer $\ell+1$ to the neuron i on layer ℓ, $w_{ij}^{(\ell)}$ be the synaptic connection weight between the neuron j on layer ℓ and the neuron i on layer $\ell-1$, and $w_{jm}^{(\ell-1)}$ connects the neuron j on layer $\ell-1$ and the neuron m on layer $\ell-2$.

Now we have a close look at $\partial\mathcal{C}/\partial w_{ij}^{(\ell)}$. According to the chain rule, we have

$$\frac{\partial \mathcal{C}}{\partial w_{ij}^{(\ell)}} = \frac{\partial \mathcal{C}}{\partial z_i^{(\ell)}} \frac{\partial z_i^{(\ell)}}{\partial w_{ij}^{(\ell)}} = \delta_i^{(\ell)} z_j^{(\ell-1)}\sigma'_{\ell,i} = z_j^{(\ell-1)}\sigma'_{\ell,i}\sum_k \delta_k^{(\ell+1)}\sigma'_{\ell+1,k}w_{ki}^{(\ell+1)}. \tag{S6}$$

The gradient term $\delta_k^{(\ell+1)} = \partial\mathcal{C}/\partial z_k^{(\ell+1)}$ is a highly coupled function of all accessible synaptic connection weights of $w_{ij}^{(\ell)}$ on the forward propagation route from $z_i^{(\ell)}$ to the output neurons. To ease the analysis, we simplify it with a numerical value or a synthetic one with no

synaptic connection weight included. Therefore, the summation term can be viewed as a simple linear function of all synaptic connection weights $w_{ki}^{(\ell+1)}$ associated with neuron i on layer ℓ, and the associated coefficient is $p\{w_{ki}^{(\ell+1)}, w_{ij}^{(\ell)}\} = z_j^{(\ell-1)}\sigma'_{\ell,i}\delta_k^{(\ell+1)}\sigma'_{\ell+1,k}$, which defines the edge weights from $w_{ki}^{(\ell+1)}$ to $w_{ij}^{(\ell)}$ on G_B. Similarly, we have the edge weight from $w_{ij}^{(\ell)}$ to $w_{jm}^{(\ell-1)}$, i.e., $p\{w_{ij}^{(\ell)}, w_{jm}^{(\ell-1)}\} = z_m^{(\ell-2)}\sigma'_{\ell-1,j}\delta_i^{(\ell)}\sigma'_{\ell,i}$. Therefore, we are able to calculate the in-degree and out-degree of $w_{ij}^{(\ell)}$, which are defined as the sum of the weights of all in-bound connections to $w_{ij}^{(\ell)}$ and the sum of the weights of all out-bound connections from $w_{ij}^{(\ell)}$, i.e.

$$\delta_{\text{in}}(w_{ij}^{(\ell)}) = z_j^{(\ell-1)}\sigma'_{\ell,i}\big[\sum_k \delta_k^{(\ell+1)}\sigma'_{\ell+1,k}\big], \tag{S7}$$

$$\delta_{\text{out}}(w_{ij}^{(\ell)}) = \big[\sum_m z_m^{(\ell-2)}\big]\sigma'_{\ell-1,j}\delta_i^{(\ell)}\sigma'_{\ell,i}. \tag{S8}$$

There are several exceptions, including the first hidden ($\ell = 1$), the last hidden ($\ell = L-1$) and the output ($\ell = L$) layers. For the output layer, we have

$$\frac{\partial \mathcal{C}}{\partial w_{ij}^{(L)}} = \sum_k \frac{\partial \mathcal{C}}{\partial z_k^{(L)}}\frac{\partial z_k^{(L)}}{\partial w_{ij}^{(L)}} = z_j^{(L-1)}(z_i^{(L)} - y_i). \tag{S9}$$

Because σ_L is softmax, no explicit relation regarding $w_{ij}^{(L)}$ can be built. It implies that no well-defined in-bound connections to $w_{ij}^{(L)}$, i.e., $\delta_{\text{in}}(w_{ij}^{(L)}) = 0$. But, we can build the connections from $w_{ij}^{(L)}$ to $w_{jm}^{(L-1)}$. It is easy to derive

$$\frac{\partial \mathcal{C}}{\partial w_{ij}^{(L-1)}} = \frac{\partial \mathcal{C}}{\partial z_i^{(L-1)}}\frac{\partial z_i^{(L-1)}}{\partial w_{ij}^{(L-1)}} = z_j^{(L-2)}\sigma'_{L-1,i}\sum_k (z_k^{(L)} - y_k)w_{ki}^{(L)}. \tag{S10}$$

From the perspective of $w_{ij}^{(L-1)}$, we get $p\{w_{ki}^{(L)}, w_{ij}^{(L-1)}\} = z_j^{(L-2)}\sigma'_{L-1,i}(z_k^{(L)} - y_k)$; from the perspective of $w_{ij}^{(L)}$, we have $p\{w_{ij}^{(L)}, w_{jm}^{(L-1)}\} = z_m^{(L-2)}\sigma'_{L-1,j}(z_i^{(L)} - y_i)$. Therefore, we get

$$\begin{aligned}
\delta_{\text{out}}(w_{ij}^{(L)}) &= \big[\sum_m z_m^{(L-2)}\big]\sigma'_{L-1,j}(z_i^{(L)} - y_i),\\
\delta_{\text{in}}(w_{ij}^{(L-1)}) &= z_j^{(L-2)}\sigma'_{L-1,i}\sum_k (z_k^{(L)} - y_k) = 0,\\
\delta_{\text{out}}(w_{ij}^{(L-1)}) &= \big[\sum_m z_m^{(L-3)}\big]\sigma'_{L-2,j}\delta_i^{(L-1)}\sigma'_{L-1,i}.
\end{aligned} \tag{S11}$$

The softmax $\sigma_L(\cdot)$ makes the output values sum up to one, i.e., $\sum_k y_k = 1$, and $\delta_{\text{in}}(w_{ij}^{(L-1)}) = 0$. Now, we examine the first hidden layer. Similar to the output layer, there is no well-defined out-bound connections for $w_{ij}^{(1)}$, $\delta_{\text{out}}(w_{ij}^{(1)}) = 0$. Setting $\ell = 1$ in Eq.(S7), we can get the in-degree of

$w_{ij}^{(1)}$

$$\delta_{\text{in}}(w_{ij}^{(1)}) = z_j^{(0)}\sigma'_{1,i}\big[\sum_k \delta_k^{(2)}\sigma'_{2,k}\big]. \tag{S12}$$

Based on our definition of the weights of G_B, when the number of layers is small, it is trivial that $\beta_{\text{eff}} = 0$. To get a non-trivial β_{eff}, we identify the minimum number of hidden layers in G_A. First, we examine a G_A with one hidden layer, i.e. $L = 2$, whose degrees are

$$\begin{aligned} \delta_{\text{in}}(w_{ij}^{(1)}) =& \ \delta_{\text{out}}(w_{ij}^{(1)}) = \delta_{\text{in}}(w_{ij}^{(2)}) = 0, \\ \delta_{\text{out}}(w_{ij}^{(2)}) =& \ \big[\textstyle\sum_m z_m^{(0)}\big]\sigma'_{1,j}(z_i^{(2)} - y_i). \end{aligned} \tag{S13}$$

Since the degrees sum up to zero, $\beta_{\text{eff}} = 0$, regardless of how many hidden neurons in G_A.

If G_A only has two hidden layers, we have the in-degrees

$$\begin{aligned} \delta_{\text{in}}(w_{ij}^{(1)}) =& \ z_j^{(0)}\sigma'_{1,i}\big[\textstyle\sum_{k=1}^{n_2}\delta_k^{(2)}\sigma'_{2,k}\big], \\ \delta_{\text{in}}(w_{ij}^{(2)}) =& \ \delta_{\text{in}}(w_{ij}^{(3)}) = 0, \end{aligned} \tag{S14}$$

and the out-degrees

$$\begin{aligned} \delta_{\text{out}}(w_{ij}^{(1)}) &= 0, \\ \delta_{\text{out}}(w_{ij}^{(2)}) &= \big[\textstyle\sum_{m=1}^{n_0} z_m^{(0)}\big]\sigma'_{1,j}\delta_i^{(2)}\sigma'_{2,i}, \\ \delta_{\text{out}}(w_{ij}^{(3)}) &= \big[\textstyle\sum_{m=1}^{n_1} z_m^{(1)}\big]\sigma'_{2,j}(z_i^{(3)} - y_i). \end{aligned} \tag{S15}$$

The total degree may be non-zero, but $\beta_{\text{eff}} = 0$ always holds. **Therefore, the minimum number of hidden layers required for a well-defined β_{eff} is three, i.e., $L \geq 4$.** We summarize the in-degrees

$$\begin{aligned} \delta_{\text{in}}(w_{ij}^{(1)}) &= z_j^{(0)}\sigma'_1(a_i^{(1)})\big[\sum_k \delta_k^{(2)}\sigma'_{2,k}\big], \\ \delta_{\text{in}}(w_{ij}^{(\ell)}) &= z_j^{(\ell-1)}\sigma'_{\ell,i}\big[\sum_k \delta_k^{(\ell+1)}\sigma'_{\ell+1,k}\big], \\ \delta_{\text{in}}(w_{ij}^{(L-1)}) &= \delta_{\text{in}}(w_{ij}^{(L)}) = 0. \end{aligned} \tag{S16}$$

and the out-degrees

$$\begin{aligned} \delta_{\text{out}}(w_{ij}^{(1)}) &= 0, \\ \delta_{\text{out}}(w_{ij}^{(\ell)}) &= \big[\sum_m z_m^{(\ell-2)}\big]\sigma'_{\ell-1,j}\delta_i^{(\ell)}\sigma'_{\ell,i}, \\ \delta_{\text{out}}(w_{ij}^{(L-1)}) &= \big[\sum_m z_m^{(L-3)}\big]\sigma'_{L-2,j}\delta_i^{(L-1)}\sigma'_{L-1,i}, \\ \delta_{\text{out}}(w_{ij}^{(L)}) &= \big[\sum_m z_m^{(L-2)}\big]\sigma'_{L-1,j}(z_i^{(L)} - y_i). \end{aligned} \tag{S17}$$

for $\forall 1 < \ell < L-1$. It is easy to derive

$$\begin{aligned}\boldsymbol{\delta}_{\text{in}}^T\boldsymbol{\delta}_{\text{out}} &= \sum_{i,j}\sum_{1<\ell<L-1} \delta_{\text{in}}(w_{ij}^{(\ell)})\delta_{\text{out}}(w_{ij}^{(\ell)}) \\ &= \sum_{i,j}\sum_{1<\ell<L-1} \big[\sum_m z_m^{(\ell-2)}\big] z_j^{(\ell-1)}\sigma'_{\ell-1,j}[\sigma'_{\ell,i}]^2\delta_i^{(\ell)}\big[\sum_k \delta_k^{(\ell+1)}\sigma'_{\ell+1,k}\big], \\ &= \sum_{1<\ell<L-1} [\mathbf{1}^T\boldsymbol{z}^{(\ell-2)}]\times\mathbf{1}^T[\boldsymbol{z}^{(\ell-1)}\odot\boldsymbol{\sigma}'_{\ell-1}]\times\mathbf{1}^T[\boldsymbol{\delta}^{(\ell)}\odot\boldsymbol{\sigma}'^2_{\ell}]\times\mathbf{1}^T[\boldsymbol{\delta}^{(\ell+1)}\odot\boldsymbol{\sigma}'_{\ell+1}].\end{aligned} \tag{S18}$$

Now, we move forward to compute the total degree

$$\begin{aligned}\mathbf{1}^T\boldsymbol{\delta}_{\text{in}} &= \sum_{ij} z_j^{(0)}\sigma'_{1,i}\big[\sum_k \delta_k^{(2)}\sigma'_{2,k}\big] + \sum_{ij}\sum_{1<\ell<L-1} z_j^{(\ell-1)}\sigma'_{\ell,i}\big[\sum_k \delta_k^{(\ell+1)}\sigma'_{\ell+1,k}\big], \\ &= [\mathbf{1}^T\boldsymbol{z}^{(0)}]\times[\mathbf{1}^T\boldsymbol{\sigma}'_1]\times\mathbf{1}^T[\boldsymbol{\delta}^{(2)}\odot\boldsymbol{\sigma}'_2] + \sum_{1<\ell<L-1}[\mathbf{1}^T\boldsymbol{z}^{(\ell-1)}]\times[\mathbf{1}^T\boldsymbol{\sigma}'_\ell]\times\mathbf{1}^T[\boldsymbol{\delta}^{(\ell+1)}\odot\boldsymbol{\sigma}'_{\ell+1}], \\ &= \sum_{1\le\ell<L-1}[\mathbf{1}^T\boldsymbol{z}^{(\ell-1)}]\times[\mathbf{1}^T\boldsymbol{\sigma}'_\ell]\times\mathbf{1}^T[\boldsymbol{\delta}^{(\ell+1)}\odot\boldsymbol{\sigma}'_{\ell+1}].\end{aligned} \tag{S19}$$

The definitions of in-degree and out-degree ensure that $\mathbf{1}^T\boldsymbol{\delta}_{\text{in}} = \mathbf{1}^T\boldsymbol{\delta}_{\text{out}}$ must hold. Let's prove it:

$$\begin{aligned}\mathbf{1}^T\boldsymbol{\delta}_{\text{out}} &= \sum_{i,j}\big[\sum_{1<\ell\le L-1}\sum_m z_m^{(\ell-2)}\sigma'_{\ell-1,j}\delta_i^{(\ell)}\sigma'_{\ell,i} + \sum_m z_m^{(L-2)}\sigma'_{L-1,j}(z_i^{(L)}-y_i)\big], \\ &= \sum_{1<\ell\le L-1}[\mathbf{1}^T\boldsymbol{z}^{(\ell-2)}]\times[\mathbf{1}^T\boldsymbol{\sigma}'_{\ell-1}]\times\mathbf{1}^T[\boldsymbol{\delta}^{(\ell)}\odot\boldsymbol{\sigma}'_\ell] \\ &= \mathbf{1}^T\boldsymbol{\delta}_{\text{in}}.\end{aligned} \tag{S20}$$

With the fact that $\boldsymbol{\sigma}'^2_\ell = \boldsymbol{\sigma}'_\ell$ for ReLU, according to Eq.(2), we have $\beta_{\text{eff}} = \boldsymbol{\delta}_{\text{in}}^T\boldsymbol{\delta}_{\text{out}}/(\mathbf{1}^T\boldsymbol{\delta}_{\text{out}})$. Specifically, we have

$$\beta_{\text{eff}} = \frac{\sum_{\ell=2}^{L-2}[\mathbf{1}^T\boldsymbol{z}^{(\ell-2)}]\times\mathbf{1}^T[\boldsymbol{z}^{(\ell-1)}\odot\boldsymbol{\sigma}'_{\ell-1}]\times\mathbf{1}^T[\boldsymbol{\delta}^{(\ell)}\odot\boldsymbol{\sigma}'_\ell]\times\mathbf{1}^T[\boldsymbol{\delta}^{(\ell+1)}\odot\boldsymbol{\sigma}'_{\ell+1}]}{\sum_{\ell=2}^{L-1}[\mathbf{1}^T\boldsymbol{z}^{(\ell-2)}]\times[\mathbf{1}^T\boldsymbol{\sigma}'_{\ell-1}]\times\mathbf{1}^T[\boldsymbol{\delta}^{(\ell)}\odot\boldsymbol{\sigma}'_\ell]}. \tag{S21}$$

Supplementary Note 3. DERIVATION OF ADJACENCY MATRIX P OF G_B

The right hand side (RHS) of Eq.(5) is a function of $W^{(\ell+1)}$, and denoted as $F(W^{(\ell+1)})$. Here we derive the strength of the impact from $W^{(\ell+1)}$ and other weights $W^{(-\ell)} = (W^{(0)}, W^{(1)}, \ldots, W^{(\ell)}, W^{(\ell+2)}, \ldots, W^{(L)})$ for building the edge dynamics. Let $W = (W^{(\ell+1)}, W^{(-\ell)})$ and $F(W) = dW^{(\ell)}/dt$. We denote $\hat{W}^{(-\ell)}$ as the current states of $W^{(-\ell)}$), $W^{*(\ell+1)}$ as an equilibrium point, and $W^* = (W^{*(\ell+1)}, \hat{W}^{(-\ell)})$. According to the Taylor expan-

sion, we linearize F at W^* and have

$$\begin{aligned} dW^{(\ell)}/dt \approx F(W^*) + \frac{\partial F(W^{*(\ell+1)}, \hat{W}^{(-\ell)})}{\partial W^{(\ell+1)}}(W^{(\ell+1)} - W^{*(\ell+1)}) \\ + \frac{\partial F(W^{*(\ell+1)}, \hat{W}^{(-\ell)})}{\partial W^{(-\ell)}}(W^{(-\ell)} - \hat{W}^{(-\ell)}). \end{aligned} \tag{S22}$$

The last term on the RHS can be cancelled out when the realizations of $W^{(-\ell)}$ take the current states of $W^{(-\ell)}$, i.e. $\hat{W}^{(-\ell)}$. The gradient is simplified as

$$dW^{(\ell)}/dt \approx F(W^*) + \frac{\partial F(W^{*(\ell+1)}, \hat{W}^{(-\ell)})}{\partial W^{(\ell+1)}}(W^{(\ell+1)} - W^{*(\ell+1)}). \tag{S23}$$

The second term $\partial F(W^{*(\ell+1)}, \hat{W}^{(-\ell)})/\partial W^{(\ell+1)} = \partial^2 \mathcal{C}(W^{*(\ell+1)}, \hat{W}^{(-\ell)})/\partial W^{(\ell)} \partial W^{(\ell+1)}$ measures how much F is affected by a unit perturbation on $W^{(\ell+1)}$, therefore can effectively captures the interaction strengths between $W^{(\ell)}$ and $W^{(\ell+1)}$. Usually, $W^{*(\ell+1)}$ are not available before the update of $W^{(\ell+1)}$, which follows the update of $W^{(\ell)}$, we use the current states of $W^{(\ell+1)}$ instead. The system can be viewed as a realization of the general Eq.(1), with linear $f(W^{(\ell)}) = F(W^*)$ and $g(W^{(\ell)}, W^{(\ell+1)}) = W^{*(\ell+1)} - W^{(\ell+1)}$. Now, we can immediately have the adjacency matrix P of G_B with $P^{(l,l+1)} = \partial^2 \mathcal{C}(W^{(\ell+1)}, \hat{W}^{(-\ell)})/\partial W^{(\ell)} \partial W^{(\ell+1)}$, $\forall 1 \leq \ell \leq L$.

Supplementary Note 4. PROOF OF THEOREM 1

The second order gradient $P^{(l,l+1)} = \partial^2 \mathcal{C}/\partial W^{(\ell)} \partial W^{(\ell+1)}$ is proposed to measure the interaction strength between $W^{(\ell)}$ and $W^{(\ell+1)}$, $\forall 1 \leq \ell \leq L$. Considering an MLP, and assume that each activation function σ_ℓ is ReLU for $\ell < L$, when G_A converges, $\boldsymbol{\nabla}_W^{(\ell)}$ vanishes, i.e., $\boldsymbol{\nabla}_W^{(\ell)} = (\boldsymbol{\delta}^{(\ell)} \odot \boldsymbol{\sigma'}^{\ell}) \boldsymbol{z}^{(\ell-1)T} = \mathbf{0}$ (Eq.S5 in Supplementary Information Supplementary Note 2). It indicates that $(\boldsymbol{\delta}^{(\ell)} \odot \boldsymbol{\sigma'}^{\ell})_i z_j^{(\ell-1)} = 0$, i.e., either $(\boldsymbol{\delta}^{(\ell)} \odot \boldsymbol{\sigma'}^{\ell})_i = 0$ or $z_j^{(\ell-1)} = 0, \forall (i,j)$. According to Eq.(S21), the numerator involves the product of terms $\boldsymbol{\delta}^{(\ell)} \odot \boldsymbol{\sigma'}^{\ell}$ and $\boldsymbol{z}^{(\ell-1)}$, which are zeros (a small constant ε is added to the denominator of β_{eff} to avoid division by zero), so $\beta_{\text{eff}} = 0$.

Supplementary Note 5. RUNNING TIME COMPARISON

We compare the running time of the proposed method in comparison to BGRN, LSV, BSV and CL. The running time is recorded in the Table S1. The CL method has a pronounced longer running time compared to other methods. The LSV and BSV method is fastest but generally gives a worse prediction compared to other methods. Our method can achieve a competitive performance and have a faster inference rate.

Supplementary Note 6. MEAN-FIELD APPROACH

We summarize the main idea of the mean-field approach developed by Gao et al.[1], and show how it is obtained[2, 3].

We consider a vertex i and the interaction term $\sum_j P_{ij} g(x_i, x_j)$ in Eq.(1), where P_{ij} is the influence j has on i. Similarly, i influences j with a weight P_{ji}. We define the in-degree $\delta_i^{\text{in}} = \sum_j P_{ij}$ and the out-degree $\delta_i^{\text{out}} = \sum_j P_{ji}$. The interaction term can be rewritten as

$$\sum_j P_{ij} g(x_i, x_j) = \delta_i^{\text{in}} \frac{\sum_j P_{ij} g(x_i, x_j)}{\sum_k P_{ik}}. \tag{S24}$$

Here the in-degrees $\boldsymbol{\delta}^{\textbf{in}}$ captures the idiosyncratic part, and the average $g(\cdot,\cdot)$ captures the network effect. The mean-field approximation is to replace local averaging with global averaging, which approximates the network impact on a vertex as nearly homogeneous. Specifically, we can get

$$\frac{\sum_j P_{ij} g(x_i, x_j)}{\sum_k P_{ik}} \approx \frac{\sum_{ij} P_{ij} g(x_i, x_j)}{\sum_{ik} P_{ik}} = \frac{\mathbf{1}^T P g(x_i, \boldsymbol{x})}{\mathbf{1}^T P \mathbf{1}}, \tag{S25}$$

where the vector $g(x_i, \boldsymbol{x})$ has the jth component $g(x_i, x_j)$. A linear operator

$$\mathcal{L}_P(\boldsymbol{z}) = \frac{\mathbf{1}^T P \boldsymbol{z}}{\mathbf{1}^T P \mathbf{1}} = \frac{\boldsymbol{z}^T \boldsymbol{\delta}^{\textbf{out}}}{\mathbf{1}^T \boldsymbol{\delta}^{\textbf{in}}} \tag{S26}$$

is defined for a weighted average of the entries in $\boldsymbol{z}$. The mean-field approximation gives

$$\dot{x}_i = f(x_i) + \delta_i^{\text{in}} \mathcal{L}_P[g(x_i, \boldsymbol{x})]. \tag{S27}$$

In the first order linear approximation, we can take the $\mathcal{L}_P$-average inside g. The average of external interactions is approximately the interaction with its average, i.e. $\mathcal{L}_P[g(x_i, \boldsymbol{x})] \approx g(x_i, \mathcal{L}_P(\boldsymbol{x}))$ and

$$\dot{x}_i = f(x_i) + \delta_i^{\text{in}} g(x_i, \mathcal{L}_P(\boldsymbol{x})), \tag{S28}$$

where $\mathcal{L}_P(\boldsymbol{x})$ is a global state. Let $x_{\text{av}} \triangleq \mathcal{L}_P(\boldsymbol{x})$. Applying $\mathcal{L}_P$ to both sides of Eq.(S28) gives

$$\dot{x}_{\text{av}} = \mathcal{L}_P[f(\boldsymbol{x})] + \mathcal{L}_P[\boldsymbol{\delta}^{\text{in}} g(\boldsymbol{x}, x_{\text{av}})]. \tag{S29}$$

According to the extensive discussion and tests in[1], the in-degrees $\boldsymbol{\delta}^{\textbf{in}}$ and the interaction with the external x_{av} are roughly uncorrelated, so the $\mathcal{L}_P$-average of the product is roughly the product of $\mathcal{L}_P$-averages. Therefore, $\mathcal{L}_P[\boldsymbol{\delta}^{\textbf{in}} g(\boldsymbol{x}, x_{\text{av}})] \approx \mathcal{L}_P(\boldsymbol{\delta}^{\textbf{in}}) \mathcal{L}_P[g(\boldsymbol{x}, x_{\text{av}})]$. Using the first order linear approximation, we take the $\mathcal{L}_P$-average inside f and g

$$\dot{x}_{\text{av}} = f(\mathcal{L}_P(\boldsymbol{x})) + \mathcal{L}_P(\boldsymbol{\delta}^{\textbf{in}}) g(\mathcal{L}_P(\boldsymbol{x}), x_{\text{av}}). \tag{S30}$$

Therefore, we have

$$\dot{x}_{\text{av}} = f(x_{\text{av}}) + \beta_{\text{eff}} g(x_{\text{av}}, x_{\text{av}}), \tag{S31}$$

where $\beta_{\text{eff}} = \mathcal{L}_P(\boldsymbol{\delta}^{\mathbf{in}})$ is the resilience metric, and its steady-state is the effective network impact x_{eff}, satisfying $\dot{x}_{\text{eff}} = f(x_{\text{eff}}) + \beta_{\text{eff}} g(x_{\text{eff}}, x_{\text{eff}}) = 0$.

Supplementary Note 7. COMPUTATIONAL COST OF THE PROPOSED FRAMWORK

We record the running time in GPU hours for the proposed NCP framework on CIFAR-10 dataset (image resolution is 32×32). The computational cost is shown in the Table S2. The NCP framework consists of two stages: in the first stage, the new model consisting of pretrained model and NCP layers with random initialization is fine tuned for 50 epochs. The pretrained model is unfrozen while NCP layers are frozen.

Supplementary Note 8. COMPARISON USING STANDARD NAS BENCHMARKS

A. LC based predictors as baselines

In addition to examine our method on classic deep learning models such as ResNet and DenseNet, we also examine the effectiveness of the NCP method on NAS-Bench-201 [4]. We randomly sample 108 architectures. The highest test accuracy of the sampled architecture is 89.16 while the lowest test accuracy is 70.09. A three-layer NCP is inserted to pretrained model in the NAS-Bench-201 search space. We use the CIFAR10 dataset as the training dataset. Each dense layer has a dimension of 128 and is followed by ReLU activation and batch normalization. We use the exactly same hyperparameters: the number of epochs is 50 and the dropout rate of the dropout layer in the NCP layers is 0.4. We use SGD optimizer and a constant learning rate of 0.001 is used. After training new model with inserted NCP for 50 epochs, we compute the β_{eff} dynamics for each epoch. We select $[t_0, t_0 + 3]$ as the prediction window to predict the validation accuracy at final epoch (i.e. $t_{\text{final}} = 50$). Bayeisan regression is used for fitting and prediction is done by extrapolation to the final epoch $|\beta_{\text{eff}}| = 0$. The typical fitting result is shown in Figure S4. Using the predicted validation accuracy, we compute the correlation bewteen the test accuracy and the predicted accuracy. The result is shown in Figure S5.

B. Training-Free NAS metrics as baselines

In addition to BSV and LSV, we compare our method with the training-free NAS methods. For the baseline methods, we use the exactly same hyperparameters as reported in the references [5–7]. Our method shows a higher correlation on NAS-Bench-201 compared to ZiCo and NASWOT. It is worth mentioning that direct comparison solely on the correlation is unfair since our method is not training free while those methods are training-free.

Figure S5 left shows the performance of the proposed method on 108 randomly sampled architectures in NAS-Bench-201 search space. We further increase the number of randomly sampled architectures to 2160 and report the performance on different number of architectures. A smaller set of architectures is the subset of larger sets of architectures. The result is shown in Figure S7. As the number of sampled architecture increases, we observe an increase in the ranking correlation.

SUPPLEMENTARY REFERENCES

[1] J. Gao, B. Barzel, and A.-L. Barabási, Nature **530**, 307 (2016).

[2] C. Jiang, J. Gao, and M. Magdon-Ismail, in *Proceedings of the Twenty-Ninth International Joint Conference on Artificial Intelligence* (2020), pp. 3307–3313.

[3] C. Jiang, J. Gao, and M. Magdon-Ismail, in *Proceedings of the AAAI Conference on Artificial Intelligence* (2020), vol. 34, pp. 131–138.

[4] X. Dong and Y. Yang, arXiv preprint arXiv:2001.00326 (2020).

[5] G. Li, Y. Yang, K. Bhardwaj, and R. Marculescu, arXiv preprint arXiv:2301.11300 (2023).

[6] M. Lin, P. Wang, Z. Sun, H. Chen, X. Sun, Q. Qian, H. Li, and R. Jin, in *Proceedings of the IEEE/CVF International Conference on Computer Vision* (2021), pp. 347–356.

[7] J. Mellor, J. Turner, A. Storkey, and E. J. Crowley, in *International Conference on Machine Learning* (PMLR, 2021), pp. 7588–7598.

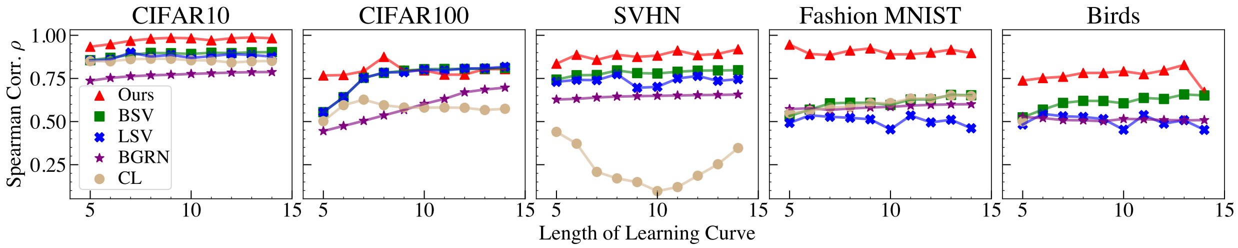

FIG. S1: A performance comparison between our β_{eff} based approach and the baselines in ranking the pre-trained models w.r.t their average accuracy over 20 runs.

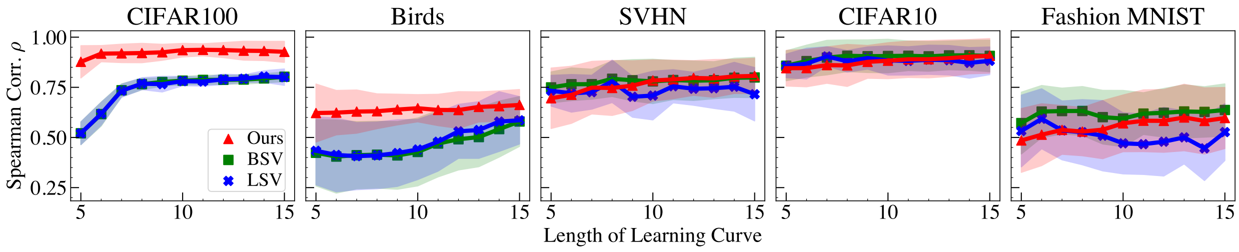

FIG. S2: A performance comparison between our β_{eff} based approach, BSV and LSV in ranking the pre-trained models w.r.t their accuracy per run (mean and standard deviation of 20 runs). BGRN and CL relies on some extra learning curves, which are impractically available in the scenario of ranking only 17 pre-trained models per run, so the related data is omitted.

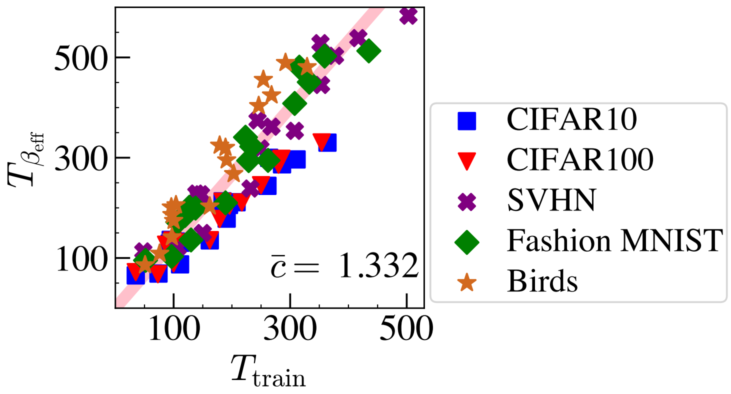

FIG. S3: Training time per epoch versus computing time for β_{eff} per epoch over all 17 pre-trained models and five datasets discussed in the main text. Each data point is associated with one pre-trained model over one dataset. The relative cost of our approach in computing β_{eff} with respect to training more epochs can be measured by $c = T_{\beta_{\text{eff}}}/T_{\text{train}}$. On average, it is $\bar{c} \approx 1.3$ (slope of the pink line).

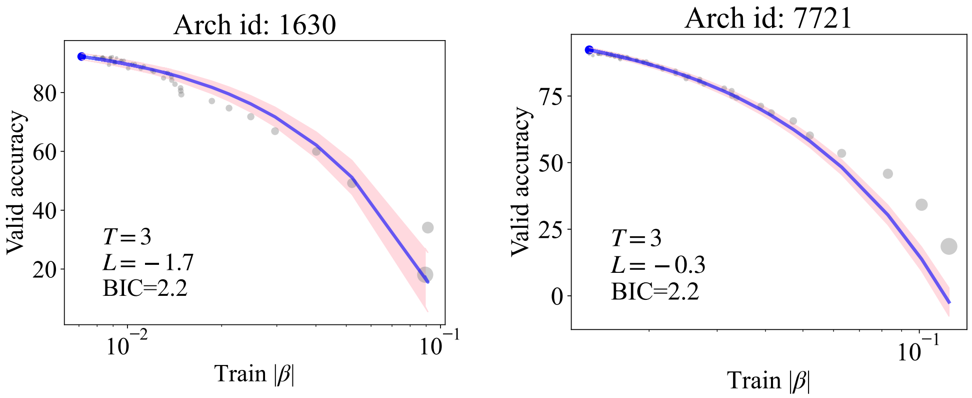

FIG. S4: Bayesian regression between the validation accuracy and $|\beta_{\text{eff}}|$ for the models in the NAS-Bench-201 search space. Left: typical fitting result for architectures with high accuracy. Right: typical fitting result for architectures with medium accuracy.

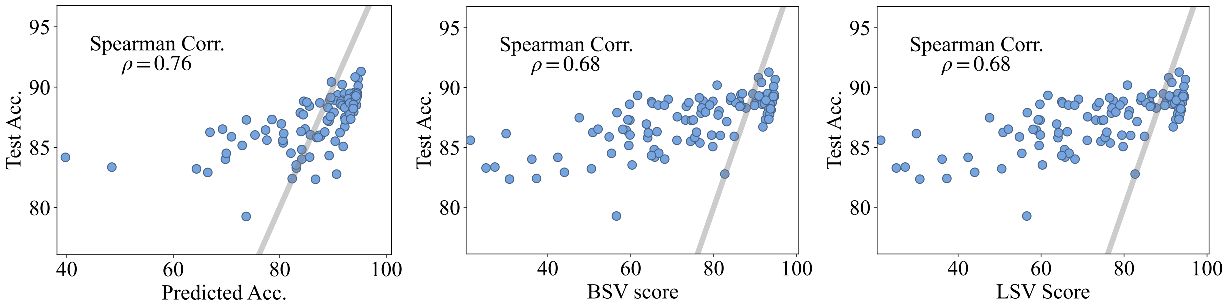

FIG. S5: Spearman correlation of predictors and test accuracy. Left: our method. Middle: BSV method. Right: LSV method.

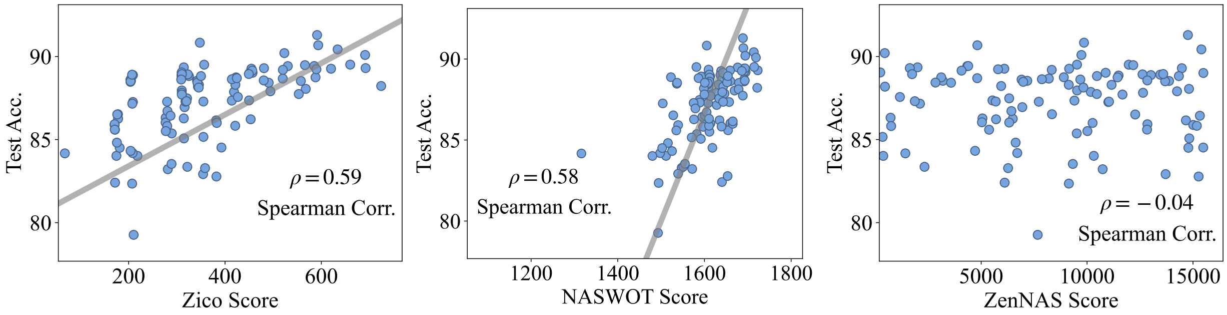

FIG. S6: Spearman correlation of architectures in the NAS-Bench-201 search space. we use a subset of architectures with performance reported in NAS-Bench-201.

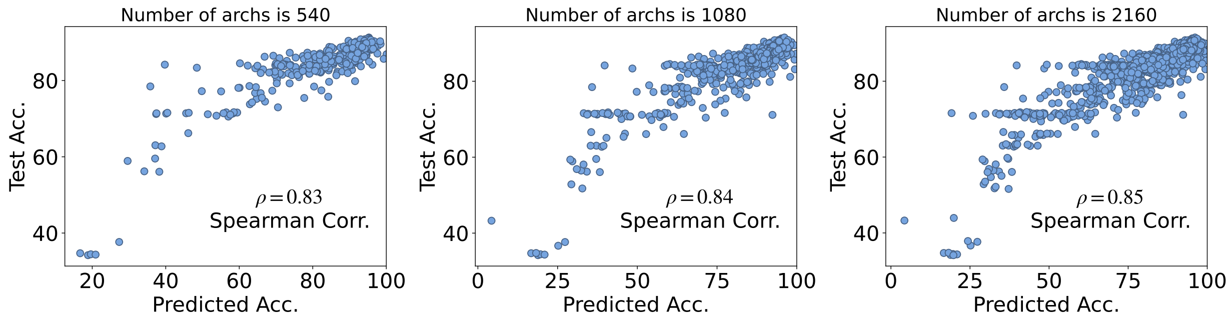

FIG. S7: Spearman correlation of the proposed method on randomly sampled architectures in the NAS-Bench-201 search space.

TABLE S1: Running time (in seconds) in learning curve prediction, including the predictor estimation (if necessary, e.g., Ours, CL and BGRN).

Dataset	Ours	BGRN	LSV	BSV	CL
CIFAR10	0.491	0.610	0.059	0.049	3966.128
CIFAR100	0.414	0.628	0.051	0.045	5256.478
SVHN	0.506	0.607	0.074	0.044	4690.507
Fashion MNIST	0.493	0.625	0.057	0.046	4552.194
Birds	0.460	0.636	0.071	0.044	4734.992

TABLE S2: Running time for the NCP framework: the first step freezes NCP probes and the pretrained model is trained to converge. The second step computes betas based on the weights at different epochs. The unit of running time is GPU hour.

Model	Fine tuning	Beta computation
ResNet18	1.32	1.18
ResNet34	1.82	1.52
ResNet50	2.88	2.20
ResNet101	3.60	3.30
ResNet152	3.96	4.23
