# Supplementary material for: Network properties determine neural network performance
Source: Nat Commun. 2024 Jul 8;15:5718. doi: 10.1038/s41467-024-48069-8 (PMC11231255; doi:10.1038/s41467-024-48069-8)
Supplement: Supplementary file 3 — Reporting Summary [file 41467_2024_48069_MOESM3_ESM.pdf]

nature portfolio

Corresponding author(s): Jianxi Gao

Last updated by author(s): Apr 16, 2024

Reporting Summary

Nature Portfolio wishes to improve the reproducibility of the work that we publish. This form provides structure for consistency and transparency in reporting. For further information on Nature Portfolio policies, see our Editorial Policies and the Editorial Policy Checklist.

Please do not complete any field with "not applicable" or n/a. Refer to the help text for what text to use if an item is not relevant to your study.
For final submission: please carefully check your responses for accuracy; you will not be able to make changes later.

Statistics

For all statistical analyses, confirm that the following items are present in the figure legend, table legend, main text, or Methods section.

n/a	Confirmed	
☐	☑	The exact sample size (*n*) for each experimental group/condition, given as a discrete number and unit of measurement
☐	☑	A statement on whether measurements were taken from distinct samples or whether the same sample was measured repeatedly
☐	☑	The statistical test(s) used AND whether they are one- or two-sided<br>*Only common tests should be described solely by name; describe more complex techniques in the Methods section.*
☐	☑	A description of all covariates tested
☐	☑	A description of any assumptions or corrections, such as tests of normality and adjustment for multiple comparisons
☐	☑	A full description of the statistical parameters including central tendency (e.g. means) or other basic estimates (e.g. regression coefficient) AND variation (e.g. standard deviation) or associated estimates of uncertainty (e.g. confidence intervals)
☐	☑	For null hypothesis testing, the test statistic (e.g. *F*, *t*, *r*) with confidence intervals, effect sizes, degrees of freedom and *P* value noted<br>*Give P values as exact values whenever suitable.*
☐	☑	For Bayesian analysis, information on the choice of priors and Markov chain Monte Carlo settings
☐	☑	For hierarchical and complex designs, identification of the appropriate level for tests and full reporting of outcomes
☐	☑	Estimates of effect sizes (e.g. Cohen's *d*, Pearson's *r*), indicating how they were calculated

Our web collection on statistics for biologists contains articles on many of the points above.

Software and code

Policy information about availability of computer code

Data collection	All training datasets (CIFAR10, CIFAR100, ImageNet) and benchmarks (NAS-Bench-201) are publicly available
Data analysis	Python

For manuscripts utilizing custom algorithms or software that are central to the research but not yet described in published literature, software must be made available to editors and reviewers. We strongly encourage code deposition in a community repository (e.g. GitHub). See the Nature Portfolio guidelines for submitting code & software for further information.

Data

Policy information about availability of data

All manuscripts must include a data availability statement. This statement should provide the following information, where applicable:

- Accession codes, unique identifiers, or web links for publicly available datasets
- A description of any restrictions on data availability
- For clinical datasets or third party data, please ensure that the statement adheres to our policy

The description and web links for publicly available datasets are included in main text.

Research involving human participants, their data, or biological material

Policy information about studies with human participants or human data. See also policy information about sex, gender (identity/presentation), and sexual orientation and race, ethnicity and racism.

Reporting on sex and gender	There are no dataset that is related to gender or sex in our work
Reporting on race, ethnicity, or other socially relevant groupings	No datasets that are socially constructed or socially relevant categorization in our work
Population characteristics	No participants are involved
Recruitment	See above
Ethics oversight	See above

Note that full information on the approval of the study protocol must also be provided in the manuscript.

Field-specific reporting

Please select the one below that is the best fit for your research. If you are not sure, read the appropriate sections before making your selection.

☐ Life sciences ☑ Behavioural & social sciences ☐ Ecological, evolutionary & environmental sciences

For a reference copy of the document with all sections, see nature.com/documents/nr-reporting-summary-flat.pdf

Life sciences study design

All studies must disclose on these points even when the disclosure is negative.

Sample size	Our work does not involve life sciences study design
Data exclusions	See above
Replication	See above
Randomization	See above
Blinding	See above

Behavioural & social sciences study design

All studies must disclose on these points even when the disclosure is negative.

Study description	This work seeks to predict the model performance through the lens of the dynamics of networks. Various datasets and deep learning models verify the superiority of the proposed method
Research sample	Training datasets being used is CIFAR10, CIFAR100, ImageNet, SVHN. We use pretrained model from Keras nad Pytorch checkpoints. Besides, we use NAS-Bench-201 benmark to obtain the model performance
Sampling strategy	All sampling process uses random sampling to avoid the potential bias
Data collection	Datasets and model checkpoint used in this research is publicly availble. Those are commonly used for deep learning research
Timing	All datasets are collected continuously without periodic gap
Data exclusions	We do not exclude any data during the model training process, i.e. we use the entire datasets
Non-participation	No participant is invovled in our work
Randomization	Randomization process is done and fixed using random seeds

Ecological, evolutionary & environmental sciences study design

All studies must disclose on these points even when the disclosure is negative.

Study description	Our work does not involve ecological, evolutionary and environmental sciences study design
Research sample	See above
Sampling strategy	See above
Data collection	See above
Timing and spatial scale	See above
Data exclusions	See above
Reproducibility	See above
Randomization	See above
Blinding	See above

Did the study involve field work? ☐ Yes ☑ No

Field work, collection and transport

Field conditions	Our work does not involve filed work, collection and transport
Location	See above
Access & import/export	See above
Disturbance	See above

Reporting for specific materials, systems and methods

We require information from authors about some types of materials, experimental systems and methods used in many studies. Here, indicate whether each material, system or method listed is relevant to your study. If you are not sure if a list item applies to your research, read the appropriate section before selecting a response.

Materials & experimental systems

n/a	Involved in the study	
☒	☐	Antibodies
☒	☐	Eukaryotic cell lines
☒	☐	Palaeontology and archaeology
☒	☐	Animals and other organisms
☒	☐	Clinical data
☒	☐	Dual use research of concern
☒	☐	Plants

Methods

n/a	Involved in the study	
☒	☐	ChIP-seq
☒	☐	Flow cytometry
☒	☐	MRI-based neuroimaging

Antibodies

Antibodies used	Our work does not involve antibodies
Validation	See above

Eukaryotic cell lines

Policy information about cell lines and Sex and Gender in Research

Cell line source(s)	Our work does not involve Eukaryotic cell lines
Authentication	See above
Mycoplasma contamination	See above
Commonly misidentified lines (See ICLAC register)	See above

Palaeontology and Archaeology

Specimen provenance	Our work does not involve Palateontology and archaeology
Specimen deposition	See above
Dating methods	See above

☒ Tick this box to confirm that the raw and calibrated dates are available in the paper or in Supplementary Information.

Ethics oversight	See above

Note that full information on the approval of the study protocol must also be provided in the manuscript.

Animals and other research organisms

Policy information about studies involving animals; ARRIVE guidelines recommended for reporting animal research, and Sex and Gender in Research

Laboratory animals	Our work does not involve animals and other research organisms
Wild animals	See above
Reporting on sex	See above
Field-collected samples	See above
Ethics oversight	See above

Note that full information on the approval of the study protocol must also be provided in the manuscript.

Clinical data

Policy information about clinical studies
All manuscripts should comply with the ICMJE guidelines for publication of clinical research and a completed CONSORT checklist must be included with all submissions.

Clinical trial registration	Our work does not involve clinical data
Study protocol	See above
Data collection	See above
Outcomes	See above

Dual use research of concern

Policy information about dual use research of concern

Hazards

Could the accidental, deliberate or reckless misuse of agents or technologies generated in the work, or the application of information presented in the manuscript, pose a threat to:

No	Yes	
☒	☐	Public health
☒	☐	National security
☒	☐	Crops and/or livestock
☒	☐	Ecosystems
☒	☐	Any other significant area

Experiments of concern

Does the work involve any of these experiments of concern:

No	Yes	
☒	☐	Demonstrate how to render a vaccine ineffective
☒	☐	Confer resistance to therapeutically useful antibiotics or antiviral agents
☒	☐	Enhance the virulence of a pathogen or render a nonpathogen virulent
☒	☐	Increase transmissibility of a pathogen
☒	☐	Alter the host range of a pathogen
☒	☐	Enable evasion of diagnostic/detection modalities
☒	☐	Enable the weaponization of a biological agent or toxin
☒	☐	Any other potentially harmful combination of experiments and agents

Plants

Seed stocks	Our work does not involve plants
Novel plant genotypes	See above
Authentication	See above

ChIP-seq

Data deposition

☒ Confirm that both raw and final processed data have been deposited in a public database such as GEO.

☒ Confirm that you have deposited or provided access to graph files (e.g. BED files) for the called peaks.

Data access links<br>*May remain private before publication.*	Our work does not involve ChIP-seq
Files in database submission	See above
Genome browser session<br>(e.g. UCSC)	See above

Methodology

Replicates	See above
Sequencing depth	See above
Antibodies	See above
Peak calling parameters	See above
Data quality	See above
Software	See above

Flow Cytometry

Plots

Confirm that:

☒ The axis labels state the marker and fluorochrome used (e.g. CD4-FITC).

☒ The axis scales are clearly visible. Include numbers along axes only for bottom left plot of group (a 'group' is an analysis of identical markers).

☒ All plots are contour plots with outliers or pseudocolor plots.

☒ A numerical value for number of cells or percentage (with statistics) is provided.

Methodology

Sample preparation	Our work does not involve flow cytometry
Instrument	See above
Software	See above
Cell population abundance	See above
Gating strategy	See above

☒ Tick this box to confirm that a figure exemplifying the gating strategy is provided in the Supplementary Information.

Magnetic resonance imaging

Experimental design

Design type	Our work does not involve magnetic resonance imaging
Design specifications	See above
Behavioral performance measures	See above
Imaging type(s)	See above
Field strength	See above
Sequence & imaging parameters	See above
Area of acquisition	See above

Diffusion MRI ☐ Used ☒ Not used

Preprocessing

Preprocessing software	See above
Normalization	See above
Normalization template	See above
Noise and artifact removal	See above
Volume censoring	See above

Statistical modeling & inference

Model type and settings	See above
Effect(s) tested	See above

Specify type of analysis: ☐ Whole brain ☐ ROI-based ☒ Both

Statistic type for inference

(See Eklund et al. 2016)

See above

Correction

See above

Models & analysis

n/a	Involved in the study
☐	☒ Functional and/or effective connectivity
☐	☒ Graph analysis
☐	☒ Multivariate modeling or predictive analysis

Functional and/or effective connectivity

See above

Graph analysis

See above

Multivariate modeling and predictive analysis

See above

This checklist template is licensed under a Creative Commons Attribution 4.0 International License, which permits use, sharing, adaptation, distribution and reproduction in any medium or format, as long as you give appropriate credit to the original author(s) and the source, provide a link to the Creative Commons license, and indicate if changes were made. The images or other third party material in this article are included in the article's Creative Commons license, unless indicated otherwise in a credit line to the material. If material is not included in the article's Creative Commons license and your intended use is not permitted by statutory regulation or exceeds the permitted use, you will need to obtain permission directly from the copyright holder. To view a copy of this license, visit http://creativecommons.org/licenses/by/4.0/
